# Effect of Sub-Lethal Exposure to Ultraviolet Radiation on the Escape Performance of Atlantic Cod Larvae (*Gadus morhua*)

**DOI:** 10.1371/journal.pone.0035554

**Published:** 2012-04-19

**Authors:** Yuichi Fukunishi, Howard I. Browman, Caroline M. F. Durif, Reidun M. Bjelland, Anne Berit Skiftesvik

**Affiliations:** Institute of Marine Research, Austevoll Research Station, Storebø, Norway; University of Hamburg, Germany

## Abstract

The amount of ultraviolet (UV) radiation reaching the earth's surface has increased due to depletion of the ozone layer. Several studies have reported that UV radiation reduces survival of fish larvae. However, indirect and sub-lethal impacts of UV radiation on fish behavior have been given little consideration. We observed the escape performance of larval cod (24 dph, SL: 7.6±0.2 mm; 29 dph, SL: 8.2±0.3 mm) that had been exposed to sub-lethal levels of UV radiation vs. unexposed controls. Two predators were used (in separate experiments): two-spotted goby (*Gobiusculus flavescens*; a suction predator) and lion's mane jellyfish (*Cyanea capillata*; a “passive" ambush predator). Ten cod larvae were observed in the presence of a predator for 20 minutes using a digital video camera. Trials were replicated 4 times for goby and 5 times for jellyfish. Escape rate (total number of escapes/total number of attacks ×100), escape distance and the number of larvae remaining at the end of the experiment were measured. In the experiment with gobies, in the UV-treated larvae, both escape rate and escape distance (36%, 38±7.5 mm respectively) were significantly lower than those of control larvae (75%, 69±4.7 mm respectively). There was a significant difference in survival as well (UV: 35%, Control: 63%). No apparent escape response was observed, and survival rate was not significantly different, between treatments (UV: 66%, Control: 74%) in the experiment with jellyfish. We conclude that the effect and impact of exposure to sub-lethal levels of UV radiation on the escape performance of cod larvae depends on the type of predator. Our results also suggest that prediction of UV impacts on fish larvae based only on direct effects are underestimations.

## Introduction

Increases in ultraviolet B (UV-B) radiation (wavelength: 280–315 nm) at the earth's surface have been observed over the past few decades and are related to depletion of the ozone layer [1], [2]. For example, in Belsk Poland, the annual sum of the daily UV-B dose has increased by 5.6±0.9% per decade over the 1979–2008 period [3]. Although the adoption of the Montreal Protocol has been successful in reducing production and consumption of some ozone destroying chemicals, elevated UV-B doses are expected to continue for several more decades until ozone returns to pre-1980 levels [4], [5]. Indeed, Arctic ozone reached an unprecedented low level (about 40% loss) at the beginning of winter to late March in 2011 [6]. Further, Nitrous Oxide (N_2_O), which is currently unregulated by the Montreal Protocol, is the single most important ozone-depleting substance today and is predicted to remain such for the rest of the 21th century. This will further delay the recovery of the ozone layer [7].

Since UV-B penetrates into the ocean's surface waters, it is regarded as an environmental stressor [8]. The depth of UV-B penetration is highly variable because factors such as turbidity, dissolved organic matter (DOM), phytoplankton concentration and suspended particles change the water's optical properties [9]. For instance, in the hyper-oligotrophic waters of the South Pacific, the depth at which UV-B radiation (at 305 nm) is reduced to 10% of the surface value (Z10%) was a maximum of 28 m [10]. In Northern European coastal waters, Z10% (at 310 nm) was 0.08–10.4 m [11].

Extensive research has been performed on the impacts of UV-B radiation on a wide variety of marine organisms such as bacteria, microalgae, phytoplankton, zooplankton, coral and fish [8]. Fish are especially vulnerable to UV-B radiation during the planktonic early life stages [12], [13], during which DNA replication and gene expression are active and adult organ systems develop during the larva-juvenile transformation. Fish larvae are also often present in the surface waters (see [14]). A large number of studies have reported deleterious effects of UV-B on fish eggs and larvae such as DNA damage [15], [16], increased mortality [12], [13], [17]–[19] malformation [20], lesion of skins, eyes and brains [12], [21], [22], retarded growth [12], [23], and immune depression [23]–[26]. The indirect effects of UV radiation on aquatic ecosystems have recently been receiving more attention (e.g. [27]). However, the indirect and sub-lethal impacts of UV-B radiation on the behavior of fish larvae and juveniles are still poorly known. Juvenile rainbow trout (*Oncorhynchus mykiss*) showed increased swimming activity and restless behavior such as rapid tail and fin movements and erratic displacements when they were exposed to UV-B radiation [28]. Vehniäinen et al. [29] reported that pike larvae (*Esox lucius*) displayed spiral swimming syndrome after a 6 hour exposure to UV-B radiation, even when the UV-B dose was sub-lethal. Juvenile coho salmon (*Oncorhynchus kisutch*) increased their shade-seeking behavior, made fewer feeding strikes, and displayed reduced agonistic behavior in the presence of UV radiation [30], [31]. To date, no studies have examined the effects of UV-B radiation on the escape behavior of fish larvae from a predator. In general, recruitment success of marine fish is determined largely by survival during their early life stages and predation is considered a major component of the mortality [32], [33]. Therefore, it is important to assess the indirect impacts of UV-B radiation on predator-prey interactions.

Atlantic cod (*Gadus morhua*) is a commercially important species of marine fish. They spawn pelagic eggs from January to May in the North Sea. Although cod larvae are broadly distributed in the water column - from the surface to the bottom (0–80 m) - they actively swim up closer to the water's surface when they start feeding [34] and are thereafter, the most abundant in the upper layers (<50 m) [35], [36]. Therefore, they are at risk of exposure to UV-B. At high latitudes, fish larvae can be exposed to UV-B for longer periods per day because of the longer daylength during their spawning season Further, the spawning grounds of the Arcto-Norwegian cod are within the region of severe ozone depletion.

Even if the damage induced by UV-B is not lethal, fish can be weakened by exposure, which might lead to reduced anti-predator performance. To examine this hypothesis, we compared the escape responses of larval cod that had been exposed to sub-lethal levels of UV-B radiation to that of unexposed controls. In addition, to see if the escape performance changes depending on the type of predator, two different predator species were used (in separate experiments): two-spotted goby (*Gobiusculus flavescens*) and lion's mane jellyfish (*Cyanea capillata*). The two-spotted goby is a semi-pelagic species found in nearshore shallow waters to about 20 m depth [37], [38]. They are visual predators that purse and suck small pelagic zooplankton [39]. Lion's mane jellyfish, a tactile predator, occur in high abundance from spring to summer in the upper water column mainly in northern coastal waters [40]. They sweep and entangle prey with their tentacles and sting and paralyze them with nematocysts. Fish larvae have been observed in their gut [40].

## Materials and Methods

### Experimental animals

Two-spotted goby *Gobiusculus flavescens* were collected using a handnet nearshore at the Austevoll Research Station, Institute of Marine Research. They were immediately transferred to the laboratory and maintained in 40 L holding tanks with moderate aeration. They were acclimatized for 3 days in the stock tank. One day before the experiment, fish were individually transferred to a small plastic container (1000 mL) so that they could be introduced into the experimental tank. Lion's mane Jellyfish *Cyanea capillata* were collected by gently scooping them from the surface water using a bucket at the Austevoll Research Station one day before the experiment. They were held in tanks without aeration. The water temperature in the stock tanks was 12°C.

Rearing techniques and protocols for cod eggs and larvae followed those detailed in [41]. Fish were handled, and the experiments conducted, under a protocol accepted by the Institutional Animal Care Committee (ID 3415).

### Ultraviolet exposure

**Figure 1:**
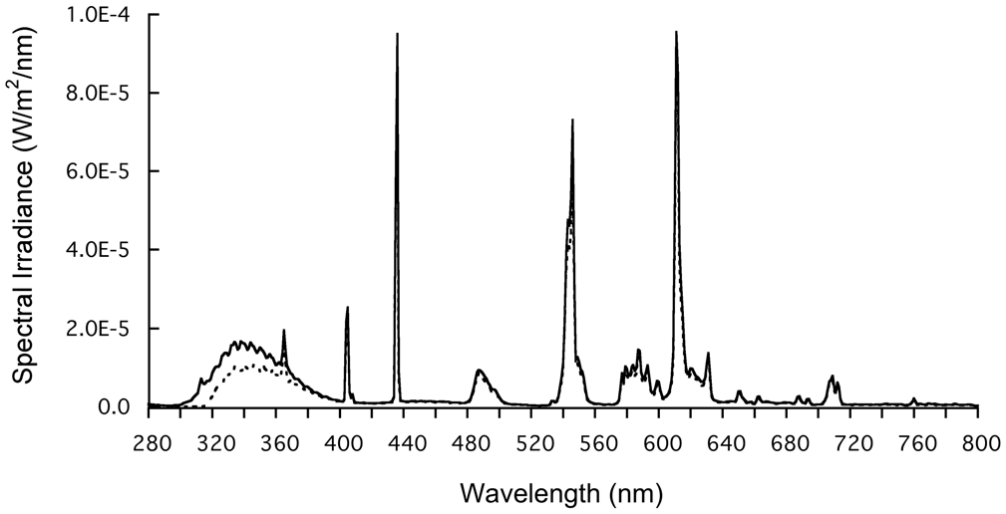
Spectral irradiance at the surface of the water in the tanks in which cod larvae (*Gadus morhua*) were exposed. Solid line denotes the UV-B treatment and dotted line denotes Control. UV-B wavelengths (280–320 nm) were blocked by Mylar-D film in Control.

Two circular exposure tanks (40 L, diameter: 45 cm; depth: 28 cm) were prepared in the laboratory. Temperature-controlled seawater (12°C) (flow-through) and moderate aeration was provided. Three UV-A lamps (UV-A 340, Q-Lab corporation, USA) and 3 fluorescent lamps (Polylux XL F36w/830, General Electric, UK) were placed alternately above the tanks at a distance of 30 cm away from the water surface. To block UV-B radiation, one of the tanks was covered with Mylar-D film (Du-Pont, USA). This was defined as a no-UV-B control treatment. Note that Mylar-D also blocks approximately 30% of UV-A, although mostly at the shorter wavelengths. The other tank received full wavelength exposure and was defined as the UV-B treatment. About 300 cod larvae were transferred from the stock tank to the exposure tank for each treatment to which they were exposed for 15 hours. During the exposure, the experimental tanks were surrounded by a black curtain. The spectral irradiance delivered to each treatment (at the water surface) was measured using a spectrophotometer (Houch & Gousego/Optronic Laboratories, OL 756, USA). Irradiance spectra are presented in Figure 1 and the total irradiances in the UV-B (280–320 nm), UV-A (320–400 nm), and photosynthetically active radiation (PAR; 400–700 nm) in both treatments are given in Table 1. A significant percentage of the cod larval population is likely exposed to UV levels of a magnitude similar to those used in our experiments (see [42]). Ambient radiation data, measured at ground level in Bergen (60°22′43″N, 5°20′33″E, University of Bergen), 22 km north of Austevoll, was obtained from the Norwegian Radiation Protection Authority (NRPA) (described in detail in [43]).

**Table 1:** Dose rate (KJm^−2^ h^−1^) and total dose (KJm^−2^) of UV-B, UV-A and PAR provided to larvae.

	UV-B (280–320 nm)		UV-A (320–400 nm)		PAR (400–800 nm)	
Treatment	Rate	Dose	Rate	Dose	Rate	Dose
UV	2.9	43.4	28.6	428.9	64.5	968.0
Control	0.4	6.2	19.1	287.2	56.2	842.8

Cod larvae (*Gadus morhua*) were exposed to two different light conditions (UV-B and Control) for 15 hours before the behavior experiments. Measurements were made at the surface of the water in the exposure tanks.

### Behavior experiment

Following the exposure, escape behavior of cod larvae from a predator was observed in a temperature-controlled room. A circular white plastic container (diameter: 25 cm) was used as an experimental tank. Aerated seawater was slowly circulated to provide enough oxygen for fish and water temperature was kept as the same as that of the holding tanks (12°C). A plastic transparent cylinder (diameter: 90 mm) was set in the middle of the tank to separate predators and larvae. A predator (either goby or jellyfish) was placed outside of the cylinder in the tank and acclimatized for 10 minutes. Ten cod larvae were randomly selected from the exposure tank and transferred to the cylinder. After 5 minutes acclimation, the cylinder was gently removed from the tank and the escape behavior of the cod larvae from the predator was recorded for 20 minutes from above using a video camera (Handycam HDR-XR 550VE, Sony, Japan). The experimental system was enclosed by a black curtain to make the visual background homogenous and eliminate the possibility of visual disturbances. The experiment was repeated 4 times for goby and 5 times for jellyfish. The order of the two treatments (control and UV-B) was alternated between trials. The size of the predators was measured with a ruler after the experiment. The mean size of goby and jellyfish was 39±3.1 mm (standard length ± SD) and 67.8±9.4 mm (bell diameter) respectively. Twenty individual cod larvae were randomly sampled from the stock tank and their standard length was measured under a binocular microscope after anesthesia with MS222. The age and standard length of cod larvae was 24 days post-hatch (dph) and 7.6±0.2 mm for the goby experiment and 29 dph and 8.2±0.3 mm for the jellyfish experiment.

### Data analysis

In the experiment with goby, escape performance was examined by analyzing video images. Escape rate from the predator, defined as total numbers of escapes divided by total numbers of attacks multiplied by 100, was calculated. Escape distance (mm) from the predator, defined as the length of a straight line from where the larva initiated the escape response from an attack of the predator to where the prey was positioned more than one second after swimming away from a predator, was also calculated. Since escape distances were estimated based on the two dimensional images displayed on the television monitor, they are shorter than the real (3-D) trajectory of larvae. Nonetheless, since they were measured in a relative manner, we maintain that the distances measured are adequate to compare the differences between treatments. Survival was calculated at the end of the experiment for both goby and jellyfish experiments.

### Statistical analysis

Chi-square tests were performed to compare escape rate and survival rate between treatments. Difference in escape distance between treatments was examined by Mann-Whitney U-test. Student's t-test was used to compare the length of larvae between two ages. All analysis were run using JMP Ver. 4.05.J, SAS Inst.

## Results

The UV-B fluence rate in the UV-B treatment (2.9 kJ m^−2^ h^−1^) was lower than that of the maximum value measured in Bergen during the summer (3.9 kJ m^−2^ h^−1^). The total UV-B dose in the UV-B treatment (43.4 kJ m^−2^) was approximately equivalent to that of 11 hours daylight exposure.

**Figure 2:**
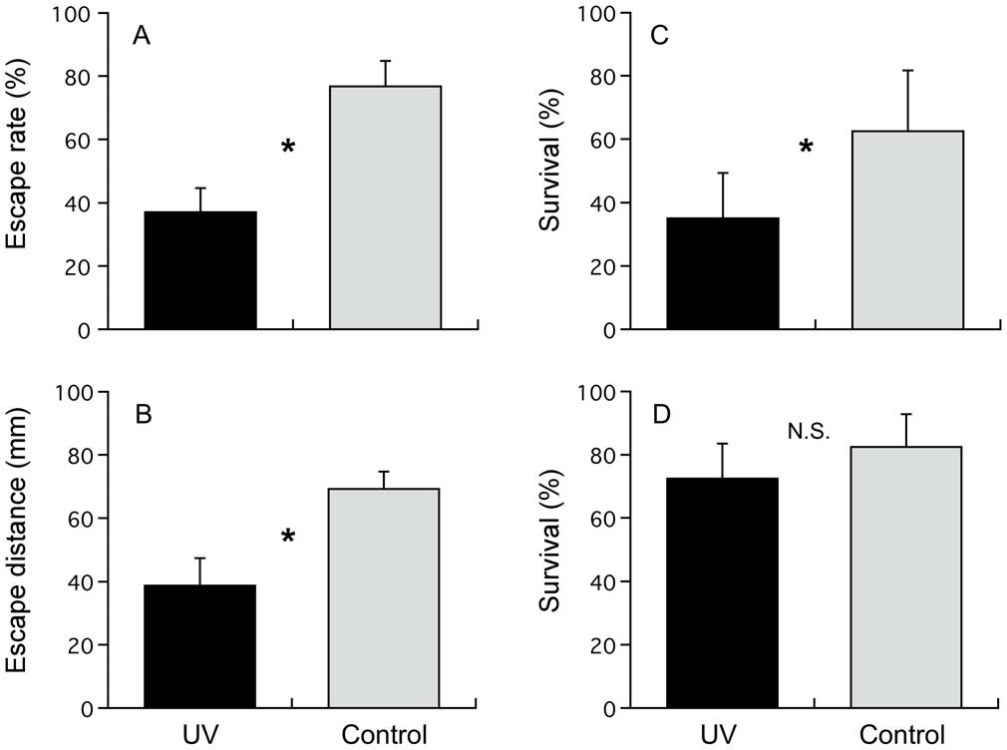
Effects of UV-B on escape performance from a predator and survival of cod larvae (*Gadus morhua*). A: Mean escape rate of cod larvae against two-spotted goby (*Gobiusculus flavescens*). B: Mean escape distance of cod larvae against two-spotted goby. C: Mean survival of cod larvae against two-spotted goby. D: Mean survival of cod larvae against lion's mane jellyfish (*Cyanea capillata*). Cod larvae in the UV-B treatment showed poorer escape rate, escape distance and survival against goby compared to that of Control. There was no significant difference between treatments was found in the survival of cod larvae against jellyfish. Asterisks indicate a significant difference between UV-B treatment and Control (*P*<0.05). Vertical bars are standard deviations.

Cod larvae in the UV-B treatment exhibited poorer escape performance against goby than did control fish. Escape rate in the UV-B treatment (Mean±SE: 36±6.7%) was significantly lower than that of control fish (75±7.0%) (*P*<0.05, Chi-square test)(Figure 2, A). Significantly lower escape distance was observed in larvae from the UV-B treatment (Mean±SE: 38±7.5 mm) compared to that of control (69±4.7 mm)(*P*<0.05, Mann-Whitney U-test)(Figure 2, B). There was a significant difference in survival (Mean±SE) between treatments (UV-B: 35±12.5%, Control: 63±16.7%)(*P*<0.05, Chi-square test) (Figure 2, C). In the experiment with jellyfish, no significant difference was observed in survival between treatments (UV-B: 66±10.8%, Control: 74±11.7%)(*P*>0.05, Mann-Whitney U-test)(Figure 2, D). The standard length of larvae between different ages was significantly different (*P*<0.05, *t*-test).

## Discussion

A sub-lethal level of UV-B exposure affected the escape performance of cod larvae when gobies were used as predators. The escape rate in UV-B treated larvae was significantly lower than that of control fish, suggesting that larvae had less possibility to avoid the predator if they had been exposed to UV-B radiation. The escape distance of cod larvae was significantly lower in the UV-B treated group compared to that of control fish. This suggests that the escape response itself was also negatively affected by UV-B exposure. Since two-spotted gobies usually form schools in the natural environment [39], UV-B-induced shorter escape distance might increase their susceptibility to attacks by successive individuals in nature. Furthermore, survival in the UV-B treatment was significantly lower than that of the control. This implies that observed negative impacts of UV-B radiation on anti-predator performance leads to higher predation mortality. Large quantities of cod eggs and larvae are consumed by pelagic predators such as herring (*Clupea harengus* L.) and mackerel (*Scomber scombrus*), and the year-strength of cod is influenced by predation [44], [45]. Thus, a sub-lethal level of UV-B exposure could indirectly increase the impact of predation on the recruitment success of cod. We suggest that the predictions of UV-B impacts on fish based only on direct lethal effects are underestimations.

Possible causes of observed lower escape capability could be energy loss by UV-B-induced physiological stress and damage. Alemanni et al. [28] exposed juvenile rainbow trout to sub-lethal level of UV-B radiation and observed stressed behavior such as rapid and erratic displacements and an increase in oxygen consumption. UV-B induced DNA damage has been reported in a variety of fish larvae such as Atlantic cod [15], [46], northern anchovy (*Englausis mordax*) [47], icefish (*Cephalus aceratus*) [16] and Japanese medaka (*Oryzias latipes*) [48]. Moreover, ATP is required for the performance of the first step of excision repair of DNA damage [49], resulting in energy loss to the recovery process (energy that could otherwise be used for other metabolic processes). Hunter et al. [12] found that sub-lethal level of UV-B exposure for a 4-day period induced lesions in the eyes of larval northern anchovy. Although we did not examine the eyes of cod larvae, there is a possibility that UV-B-induced eye damage reduced their ability to recognize predators, leading to significantly lower escape rates.

When lion's mane jellyfish were used as predators, escape behavior (swimming away continuously from the predator at much higher speed than the background swimming speed), which was seen in the experiment with goby, was not observed in either UV-B or control treatments. In addition, there was no significant difference in survival between treatments. These results suggest that a sub-lethal level of UV-B did not affect the susceptibility of cod larvae to lion's mane jellyfish predators. Jellyfish entangle prey with their tentacles and sting them with nematocysts to subdue and capture them. Lion's mane jellyfish nematocysts deliver strong venom: a neurotoxin, which paralyzes prey immediately [50]. The venom of the fishing tentacles of lion's mane jellyfish was more toxic (threefold; >30 cm, 20-fold; >20 cm) than that of moon jellyfish *Aureria aurita* of equivalent size classes at a concentration of 10 µg ml^−1^ [51]. Båmstedt et al. [52] reported that lion's mane jellyfish (bell diameter: 60–170 mm in their experiment; 67.8±9.4 mm in our experiment) could catch and ingest fish larvae and small fish (total length: 20–70 cm) much larger than those used in this experiment. Therefore, it is likely that larvae were captured irrespective of their viability or escape responses.

We did not expose the predators themselves to UV radiation. Therefore, we are unable to assess the possibility that UV also affects the feeding efficiency of gobies and jellyfish. Nonetheless, the present study indicates that sub-lethal levels of UV-B radiation increased vulnerability of cod larvae to predation by visual predators, but not to tactile predators. This result is consistent with reports for amphibians. Romansic et al. [53] demonstrated that UV-B radiation caused increased susceptibility of Cascades frog (*Rana cascadae*) larvae to predation by rough-skinned newts (*Taricha granulosa*).

Mylar-D film absorbs all UV-B radiation and reduces UV-A by approximately 30% (mainly at the shorter wavelengths). Therefore, the dose rate and total dose of UV-A radiation in the no UV-B control group was approximately 30% lower than that in the UV-B treatment. We cannot evaluate to what extent this difference in UV-A exposure contributed to our results, (because we did not have an exposure treatment with neither UV-B nor UV-A). However, we consider the probability of this to be low because earlier work demonstrates that UV-A exposure alone does not affect mortality, rate of development, pigmentation, size nor DNA damage in cod eggs and larvae [13], [18], [54]. Further, for organisms possessing photorepair mechanisms, UV-A plays a beneficial role in DNA repair [20], [47], [54].

Fish larvae have the ability to repair DNA damage caused by UV-B radiation [20], [47], [54]. However, it takes time to recover from the UV-B-induced abnormal behavior. In the case of pike larvae (*Esox lucius*), the inability to swim straight lasted for 1 week after 3 hours of a sub-lethal level of UV-B exposure on two consecutive days [29]. Cod larvae had much poorer capacity for DNA photorepair than did that of anchovy larvae and their DNA damage was often carried over into the next day, which can lead to a greater multi-day accumulation [54]. Thus, even short-term exposure to sub-lethal levels of UV-B could increase vulnerability to visual predators for periods of time much greater than the exposure itself.
